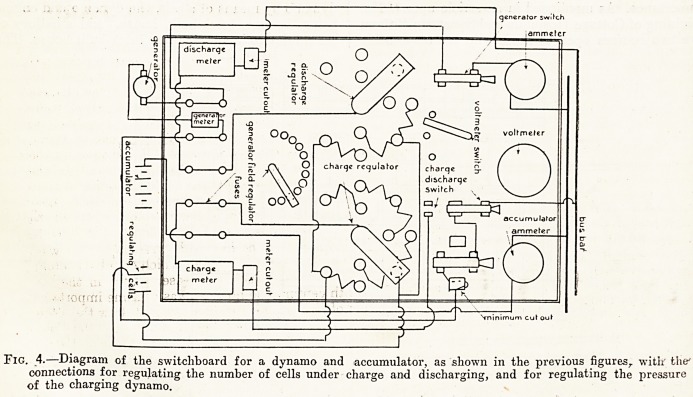# Electrical Plant for Cottage Hospitals

**Published:** 1913-03-08

**Authors:** 


### PRACTICAL POINTS.

(Criticism and Suggestions Invited.)

### Electrical Plant for Cottage Hospitals.

As the use of electricity is becoming more and more general in hospitals, and as cottage hospitals are situated very often in somewhat out-of-the-way places, the question of the use of electricity in them and how it can best be arranged is of some importance. As mentioned in an article upon "The Lighting of Cottage Hospitals and Sanatoria," there are two courses open to those who are responsible for the management of cottage hospitals: viz. taking current from the county electrical power station, if there is one, or generating current for themselves by means of an oil engine, dynamo, and accumulator. The former is by far the best arrangement, as it gives less trouble and usually costs less. If, however, it is decided to generate current upon the ground it may be done conveniently, and figs. 1, 2, and 3 are diagrams of the arrangement necessary.

Fig. 1.—Sectional drawing showing dynamo and oil engine in engine house, suitable for a cottage hospital.

As will be seen, there is an oil engine driving a dynamo by means of a belt, the dynamo and engine being in one brick-built house, and a battery of accumulators in an adjoining brick-built house. The accumulators are best placed on stands provided for them, in rows one above the other, so arranged that the attendant can walk right round them. As will be seen by the diagram, if the accumulator cells are placed in two rows horizontally, and two or more

Fig. 2.—Vertical section of accumulator house, with batteries on shelves, suitable for a cottage hospital. A transverse vertical section of the engine room is shown on the right.

rows, as may be necessary, vertically, the attendant in walking round can see any particular cell, and can take measures to put it right if it is not working properly. The reason for the accumulator being in a separate house by itself is that during the process of charging the accumulators a certain amount of hydrogen gas is given off. This is bad for machinery and for human beings, and, therefore, the battery is best confined to its own house, which should be well ventilated.

A switchboard is required for regulating the accumulator cells, converting one of the oxides of lead with which the lead plates are pasted into a higher oxide, and reducing the oxide with which the other plates are pasted to spongy lead. While this is going on a certain amount of heat is liberated in the liquid electrolyte, the dilute sulphuric acid, causing evaporation, and also a certain amount of electrolysis takes place, giving rise to the evolution of hydrogen in small quantities. It is possible to collect a small quantity of hydrogen in a test tube at the surface of any one of the cells during charge

Fig. 3.—Sectional plan of accumulator house and engine room, showing the batteries in their own building, and the engine, dynamo, etc., in a separate building adjoining

Fig. 4.—Diagram of the switchboard for a dynamo and accumulator, as shown in the previous figures, with the connections for regulating the number of cells under charge and discharging, and for regulating the pressure of the charging dynamo.

current during the time the accumulators are being charged and during the time they are furnishing current to the building. It will be remembered that the current from the dynamo passes into the and to ignite it in the usual way. It is usual to charge the battery during the day, and to take current from it during the night. It is quite easy, however, to arrange that current can be taken from the plant during the day, while it is being charged, by means of proper arrangements on the switchboard.

Two or three points are necessary to be remembered in connection with the use of accumulators. While they are being charged, the pressure of each cell is 2½ volts, and, consequently, a battery of, say, fifty cells will require a total pressure of about 130 volts from the dynamo to charge them. (The extra 6 volts is required to overcome the internal resistance of the accumulator cells.) When the cells are discharging they commence with a pressure of about 2 volts per cell, and this gradually lessens. Immediately after the charge is complete the cells will have a pressure of about 2.2 volts per cell, but the extra .2 volts is quickly lost. It is due to the presence of the gases that are liberated by the charging current, which are soon either dissolved by the electrolyte or escape into the atmosphere. As the pressure furnished by the accumulators commences to fall from the time that the charging ceases, it is necessary to have a few extra cells to bring into service, in order to keep the pressure approximately uniform as the main battery fails. These extra cells being only used during the later portion of the evening are not generally as much run down at the end of the night as the main battery, and, therefore, do not require so much charging next day. To meet these requirements it is usual to have a switch of the form shown in fig. 4, by which additional cells may be added as desired, and the number of cells under charge may also be regulated. Fig. 4 shows the usual arrangement of a switchboard for a plant of this kind, the regulating switch being shown in the middle of the board. There are automatic regulating switches upon the market, which bring additional cells into operation as the pressure falls. When it is required to use the current while the battery is being charged, another switch is necessary, regulating the pressure that is being taken off from the service. Thus, if the lamps in use in the hospital work with a pressure of 100 volts, the pressure at which the current is taken off from the combined dynamo and accumulator must only be also 100 volts or a little over, the few extra volts being provided to overcome the resistance of the wires between the accumulator and the lamps. As mentioned above, during charge, the pressure between the terminals of the accumulator will be 130 volts, and may even be more if some of the regulating cells are being charged. It is easy, however, to make a connection to a point in the battery at which the required pressure will be obtained. Thus, taking the pressure of the cells as 2½ volts, if a connection is made at the fortieth cell from one end, the required pressure will be obtained. It will be a little over 100 volts, because of the pressure absorbed in overcoming the resistance of the cells, but this extra pressure will be useful in overcoming the resistance of the cables and wires leading to the lamps.

Having the electrical plant on the ground, or a supply of electricity, it may be used for a number of other purposes besides lighting. Fans are of great service in warm weather in hospitals, and are always of use in carrying off unpleasant odours. Heating appliances worked by electricity are also very convenient, and there are a number of other apparatus that can be conveniently worked by electricity.

## Figures and Tables

**Fig. 1. f1:**
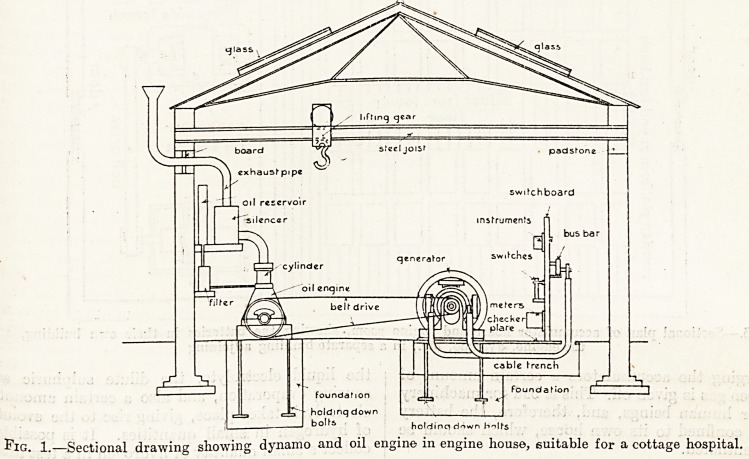


**Fig. 2. f2:**
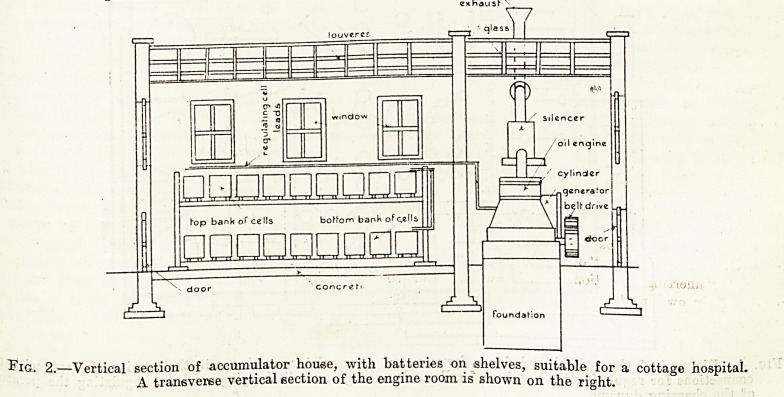


**Fig. 3. f3:**
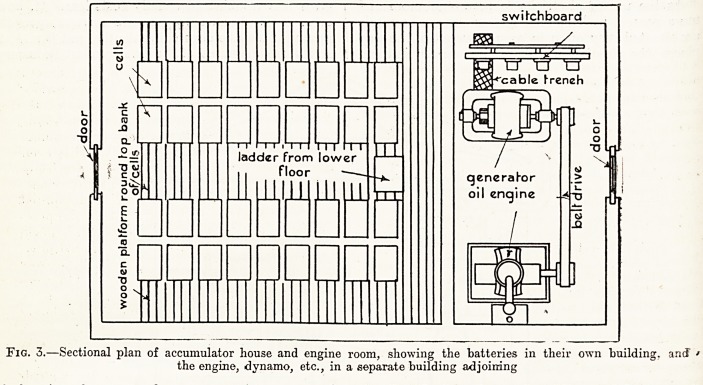


**Fig. 4. f4:**